# Measuring gait parameters from a single chest-worn accelerometer in healthy individuals: a validation study

**DOI:** 10.1038/s41598-024-62330-6

**Published:** 2024-06-17

**Authors:** N. Camerlingo, X. Cai, L. Adamowicz, M. Welbourn, D. J. Psaltos, H. Zhang, A. Messere, J. Selig, W. Lin, P. Sheriff, C. Demanuele, M. Santamaria, F. I. Karahanoglu

**Affiliations:** Pfizer, Inc., Cambridge, MA USA

**Keywords:** Biomedical engineering, Health care, Medical research

## Abstract

Digital health technologies (DHTs) are increasingly being adopted in clinical trials, as they enable objective evaluations of health parameters in free-living environments. Although lumbar accelerometers notably provide reliable gait parameters, embedding accelerometers in chest devices, already used for vital signs monitoring, could capture a more comprehensive picture of participants’ wellbeing, while reducing the burden of multiple devices. Here we assess the validity of gait parameters measured from a chest accelerometer. Twenty healthy adults (13 females, mean ± sd age: 33.9 ± 9.1 years) instrumented with lumbar and chest accelerometers underwent in-lab and outside-lab walking tasks, while monitored with reference devices (an instrumented mat, and a 6-accelerometers set). Gait parameters were extracted from chest and lumbar accelerometers using our open-source Scikit Digital Health gait (SKDH-gait) algorithm, and compared against reference values via Bland–Altman plots, Pearson’s correlation, and intraclass correlation coefficient. Mixed effects regression models were performed to investigate the effect of device, task, and their interaction. Gait parameters derived from chest and lumbar accelerometers showed no significant difference and excellent agreement across all tasks, as well as good-to-excellent agreement and strong correlation against reference values, thus supporting the deployment of a single multimodal chest device in clinical trials, to simultaneously measure gait and vital signs.

**Trial Registration**: The study was reviewed and approved by the Advarra IRB (protocol number: Pro00043100).

## Introduction

Gait is an integral part of almost all activities of daily living and, as such, it represents an indicator of overall wellbeing^1,2^. In turn, gait assessment is an essential tool for clinical applications, including but not limited to evaluating the status and the progression of numerous disorders (spanning from neuromuscular diseases, such as Duchenne muscular dystrophy and Friedreich ataxia, to cardiometabolic diseases, such as heart failure and obesity)^3,4^, predicting falls in older adults^5^, assessing life satisfaction^6^, and predicting patients’ survival^7^.

Traditionally, gait is assessed during episodic in-lab visits, by means of observational scales (e.g., the Unified Parkinson Disease Rating Scale, the Scale for the Rating and Assessment of Ataxia, the Expanded Disability Status Scale), and performance tests (e.g., 6-min walking test, Short Physical Performance Battery, Timed Up and Go). While these assessments are designed to provide standardized measures of specific aspects of gait, they cannot simultaneously capture every clinically relevant aspects of this complex process, including precise spatio-temporal characteristics such as gait speed, cadence, symmetry, and swing time. For example, the 6-min walking test only measures a distance, but it cannot capture the distribution of gait speed across the strides performed during the task, which could reveal complementary information about the quality of the walking pattern. Furthermore, the validity, reliability, and objectivity of these traditional assessments is increasingly being questioned, given their sporadic administration, the clinical environment constraints (such as a reduced space, limited time), and the intrinsic subjectivity of the questionnaires, which might be influenced by potential cognitive impairments^8,9^. The widespread uptake of digital health technologies (DHTs), such as wearable accelerometers, has the potential to improve the objectivity and reliability of gait assessment by enabling continuous remote monitoring at high frequency^10^. Firstly, DHTs allow measuring gait in free-living conditions, i.e., within individuals’ own environment, thus minimizing their travel to sites, and potentially improving their engagement. This represents an advantage especially for participants requiring assistance, including children, the elderly, and individuals with physical/cognitive impairments, as well as for participants living in remote locations, who can benefit from DHTs and be enrolled in decentralized clinical trials (DCT)^11^. Additionally, remote monitoring of gait has prognostic and diagnostic implications in clinical care^12^. Patients living in remote areas or with functional limitations that make travel difficult could be assessed via telehealth coupled with remote monitoring using DHTs in clinical practice to monitor their response to treatment and overall health. While supervised in-lab assessments are associated with the well-known risk of the Hawthorne effect^13,14^, free-living gait measurements are more representative of individuals’ physiological conditions, as far as they are collected over multiple days/weeks as necessary, in order to limit the impact of confounding factors (such as seasonality, temporary illnesses, socio-economic status, weekend variability, etc.) on the gait metrics. Finally, DHTs can enable simultaneous capture from multiple modalities (e.g., accelerometer, electrocardiogram (ECG), temperature), thus reducing participants’ and patients’ burden of wearing multiple devices, whilst enhancing the accuracy and speed of clinical interpretations^15^. In summary, DHTs can expedite the planning and conduct of participant-centric DCT, can improve diversity and inclusion in clinical trials, and facilitate patient access to clinical care.

Amongst the gait-related parameters that can be collected using DHTs, gait speed (sometimes referred to as stride velocity) is considered the most powerful biomarker of mobility^16^. Longitudinal assessment of gait speed allows clinicians to monitor the progression of a myriad of diseases including stroke, multiple sclerosis, and Parkinson’s disease^17^. In addition, the European Medicine Agency has recently qualified the 95th percentile of stride velocity as a primary endpoint in clinical trials involving patients with Duchenne muscular dystrophy, given the supportive data and evidence of its clinical meaningfulness, representing an important milestone in the adoption of wearable accelerometers in clinical trials^18^.

A crucial aspect in the deployment of wearable accelerometers in clinical trials is related to their form factor. There are various types of wearable accelerometers currently available on the market, such as lumbar bands, wrist watches, chest patches, rings, insoles, and clothes^19^. The selection of the appropriate body location depends on several aspects, including the target population (as some body locations might not be appropriate for children or for the elderly), the specific endpoints of interest (e.g., to prioritize the capture of upper or lower body movements), and user preference (which might impact compliance). One of the most popular choices to measure gait from wearable accelerometers is the lumbar belt, as it is stable and tends to experience less movement compared to other body parts (such as wrist or ankle), resulting in reduced chances of accidental bumps or interference, and an accurate tracking of gait^20,21^. Nonetheless, a chest accelerometer could be more advantageous for certain applications, such as those involving posture correction^22,23^, prediction of falls^24^, and postural sway assessments^25^. In addition, chest-worn single lead ECG patches are increasingly being used in clinical practice to continuously monitor vital signs over a period of weeks, including cardiac rhythm and respiratory rate, replacing the more cumbersome and shorter battery life Holter monitors^26,27^. Many of these new single-lead ECG patches have built-in accelerometry which may be leveraged to derive information about gait and walking ability. The deployment in clinical trials of multimodal DHTs capturing both gait and vital signs could provide valuable insights into patients’ health, in terms of physiological and physical functioning, while removing participants’ burden of wearing two or more different devices (e.g., a chest patch to measure electrocardiogram (ECG), and a lumbar belt to measure gait).

Notably, the analytical validation of digital endpoints collected from DHTs in comparison to reference (and usually non-scalable) devices is an indispensable requisite for their deployment in clinical trials^28^. Despite the great potential of multimodal DHTs, the validation of gait parameters from chest accelerometers has been minimally explored^29,30^. In this paper, we aim to assess the validity of gait parameters measured from a chest accelerometer; specifically, we employ our in-house built state-of-the-art algorithm for gait analysis, namely the Scikit Digital Health gait (SKDH-gait) algorithm^31^, already validated for a lumbar-worn accelerometer^13^, and assess its performance when applied to a chest accelerometer for healthy individuals, during multiple walking tasks, simulated daily activities, and naturalistic walking.

## Methods

### Participants

Twenty healthy individuals (13 females, mean ± sd [min–max] age: 33.9 ± 9.1 [25–61] years, BMI: 24.4 ± 4.7 [19.1–33.6] kg/m^2^, 13 identifying as white, 7 as Asian, and 3 with Hispanic or Latino ethnicity) gave informed consent and took part to the study. According to the eligibility criteria, participants did not have any significant health problem, as determined by the study physician during medical history intake. In addition, they did not have implanted medical devices and were not participating in other studies involving digital devices.

### Study design

Participants underwent two instrumented visits each lasting about two hours, performed about 7 days apart. The visits were completed at the Pfizer Innovation Research (PfIRe) Laboratory, in Cambridge, Massachusetts, USA.

During each visit, participants were instrumented with six wearable inertial devices (Opal, APDM Inc., Portland, Oregon) worn on the chest (sternal position), lumbar (L4 position), and bilaterally on the wrists and feet. The devices recorded data from three-axis accelerometer, gyroscope, and magnetometer, at sampling rate of 128 Hz. From now on, this set of wearable devices will be referred to as APDM 6-sensor set.

Participants were asked to complete in-lab walking tasks (at three self-paced walking speeds), in-lab and outside-lab simulated free-living activities with numerous walking bouts, mimicking real-life behavior. The tasks are described below, in the order they were completed:

- *In-lab walking task at self-paced speed*: Participants walked on a 20-feet instrumented gait mat (GAITRite, CIR Systems Inc., Franklin, New Jersey, USA) at normal, slow, and fast self-paced speeds, while instrumented with the APDM 6-sensor set. For each speed, they were asked to perform three laps.
- *In-lab simulated activities*: Participants were asked to move freely inside the lab during two sessions of 7-min duration, while instrumented with the APDM devices, to mimic naturalistic walking and sit-to-stand activities. They performed one or more of the following 6 daily-life activities randomized to a dice roll: (1) sitting and write; (2) sitting and rest; (3) toss an object, pick up and come back; (4) move an object; (5) hang a coat; (6) turn a switch. The dice could be rolled multiple times, until the end of the session duration.
- *Outside-lab activities*: Participants were asked to move freely for 20 min outside the lab, while instrumented with the APDM devices, followed by study team escort.

The study was reviewed and approved by the Advarra IRB (protocol number: Pro00043100). All research was performed in accordance with relevant guidelines/regulations, including data privacy and Declaration of Helsinki.

### Gait endpoints extraction

The test and reference devices used in each task, and the algorithms used to extract gait endpoints are summarized in Table 1.

**Table 1:** Summary of test and reference devices deployed for each task, with the respective algorithms used to extract gait endpoints.

Task	Test device (algorithm)	Reference devices (algorithm)
In-lab walking at self-paced speed	Chest device (SKDH-gait)	Gait mat (PKMAS) APDM 6-sensor set (APDM algorithm) Lumbar device (SKDH-gait)
In-lab simulated activities	Chest device (SKDH-gait)	APDM 6-sensor set (APDM algorithm) Lumbar device (SKDH-gait)
Outside-lab activities	Chest device (SKDH-gait)	APDM 6-sensorset (APDM algorithm) Lumbar device (SKDH-gait)

In this study, the APDM chest device was considered as a standalone test device, from which gait endpoints were derived using SKDH-gait (v 0.9.7), an in-house built, open-source algorithm implemented in Python^31^. The algorithm first uses a pre-trained classification model to predict gait bouts from various acceleration signal features extracted from 3 s windows. Consecutive 3 s gait bouts are aggregated together to form longer gait bouts. Then, for each bout, SKDH-gait uses a wavelet-based approach to detect heel strike and toe off events during a gait cycle^32^. Finally, an inverted pendulum model is employed to calculate spatial gait features^33^.

Gait endpoints were derived from the gait mat, reference device for the in-lab walking tasks, using an external software, namely ProtoKinetics Movement Analysis Software (PKMAS), from ProtoKinetics LLC (Havertown, PA, USA).

Gait endpoints from the APDM 6-sensor set, reference device for all tasks, were also derived using APDM proprietary algorithms^34^.

In a previous study, SKDH-gait applied to a lumbar accelerometer was validated by comparison against reference devices (i.e., GAITRite gait mat and APDM 6-sensor set) in a healthy volunteers study conducted in the PfIRe lab^13^. Therefore, in this study, we considered the APDM lumbar device as a standalone reference device for all tasks as well. Gait endpoints from the lumbar device were also derived using the same version of SKDH-gait (v 0.9.7).

The following gait endpoints were derived from all devices and considered for comparison during the in-lab and outside-lab assessments: (1) gait speed (m/s), (2) cadence (steps/min), (3) stride length (m), (4) stride time (s). In addition, the 95th percentile of gait speed (m/s) was derived from in-lab and outside-lab activities only, since the higher number of steps allows for a more robust estimation.

For each gait endpoint, the median value was extracted per task and visit. Then, the average across the two visits was computed, thus obtaining a single value per participant and task.

### Statistical analysis

The agreement between the average endpoints obtained from chest device and reference devices was assessed by the intra-class correlation coefficient (ICC, type 2-way random effects, absolute agreement), according to the benchmarks reported in^35^, where ICC ≤ 0.4 indicates “poor”, 0.4–0.59 “moderate”, 0.6–0.74 “good”, and 0.75–1 “excellent” agreement. To quantify the differences between the average endpoints obtained from chest device and reference devices, the following error metrics were computed: mean difference (or bias), mean absolute difference, and mean percent error. In addition, Bland–Altman plot with 95% limits of agreement (computed as average difference ± 1.96 standard deviation of the difference), and scatter plots of the average endpoints obtained from chest device and reference devices were used to visualize the agreement. Finally, the Pearson’s correlation coefficient (R) was computed.

The effect of the device and the task, and the interaction between device and task was investigated by performing a mixed model regression analysis. The model included the device, the task, and their interaction as fixed effects, while subject was modelled as a random intercept. Subject’s age was included as a covariate. The Restricted Maximum Likelihood (REML) approach was used to obtain unbiased estimates of variance parameters. ANOVA with F-test was performed to test the overall significance of the fixed effects on the error between devices. In case of significant effects (defined as *P* < 0.05), post-hoc tests were performed to assess the pairwise differences.

To ensure that measurements from SKDH-gait applied to the chest device are stable over time, and that the device can consistently reproduce the same results over time, a test–retest reliability analysis over gait endpoints collected during the two visits was conducted. Results are presented in terms of ICC and Pearson’s correlation coefficient for both the in-lab walking tasks and the in-lab simulated and outside-lab activities, and are reported in the Supplementary Material.

To compare the chest and lumbar locations in terms of SKDH-gait-detected walking bouts, a paired t-test was performed to assess differences between the number and duration of walking bouts, and the total walking time detected by SKDH-gait run on chest and lumbar devices.

All statistical analyses were performed in R (v. 3.4.2) with the following main packages: “lme4” for linear mixed-effect regression, “lmerTest” for post-hoc tests, “car” for type-III ANOVA, “BlandAltmanLeh” for Bland–Altman plots, and “psych” for ICC.

## Results

### Agreement of gait endpoints derived from chest device and reference devices during in-lab walking tasks

In this section, the agreement between gait endpoints collected with SKDH-gait run on the chest device, and those collected from reference devices; i.e., the gait mat and the APDM 6-sensor set, during in-lab walking tasks at self-paced speeds is discussed.

For one participant, gait data from the gait mat for the in-lab walking task at slow self-paced speed, during the second visit were not recorded. For this participant, task, and device, only data collected during visit 1 were considered.

**Figure 1:**
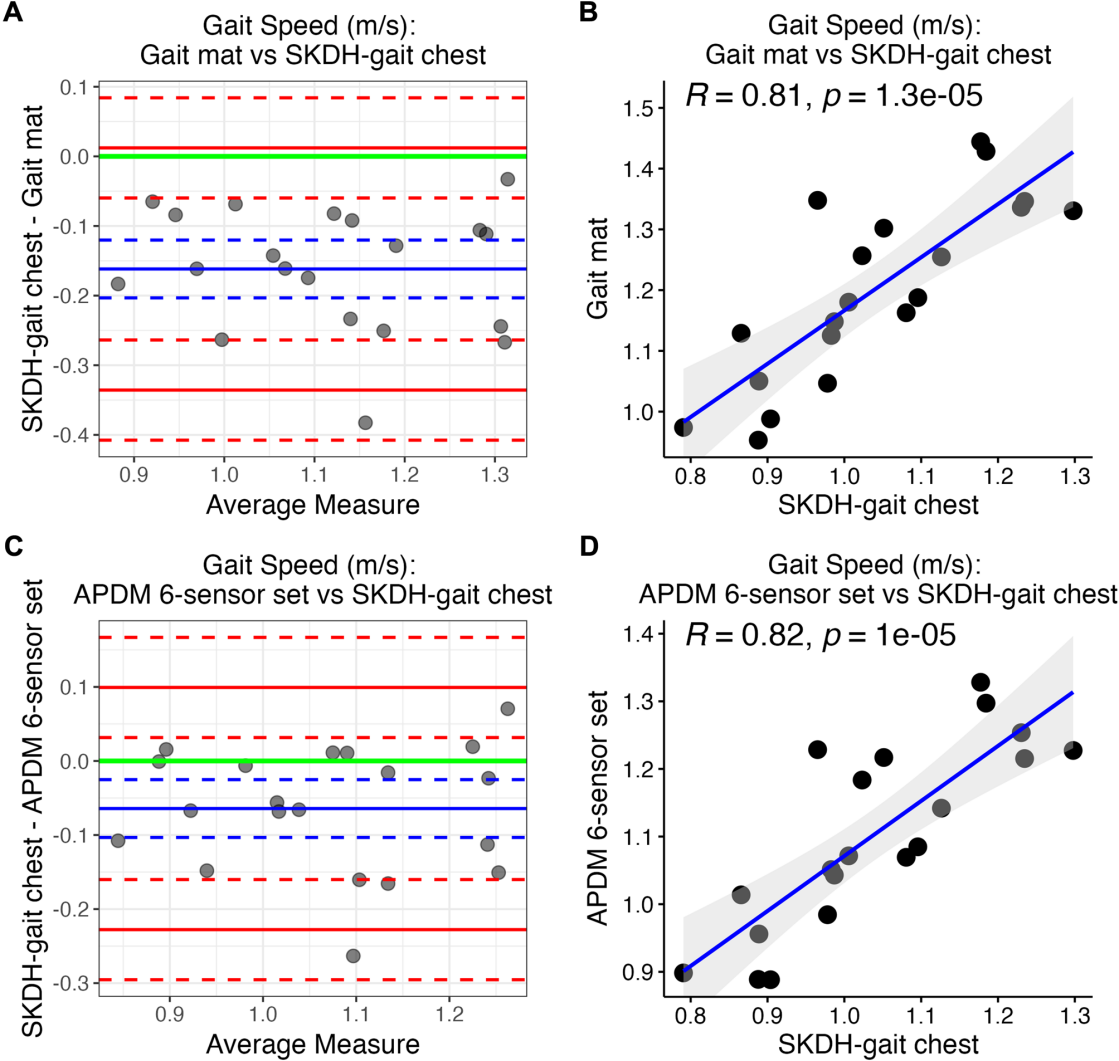
Assessment of gait speed during the walking task at normal speed. Assessment of gait speed estimated from SKDH-gait applied on the chest device against the instrumented gait mat (panels **A**, **B**), and the APDM 6-sensor set (panels **C**, **D**), during in-lab walking tasks at normal self-paced speed. Bland–Altman plots (panels **A**, **C**) show a mean bias (SKDH-gait chest—gait mat = − 0.162 m/s; SKDH-gait chest—APDM 6-sensor set = − 0.064 m/s) consistent across the measurement range (blue solid line) and narrow LoA (red solid lines), for both the reference devices. Corresponding 95% confidence intervals are in dashed lines, while green solid line represents the 0-threshold. Scatter plots (panels **B**, **D**) show highly correlated measurements for both the reference devices. Regression line (solid blue line) with 95% confidence interval (shaded gray area) are depicted, and the Pearson’s R with the related *p*-value are annotated in the figure. *LoA* Limit of Agreement.

Error metrics, Pearson’s R, and ICC are reported in Table 2, for gait speed. Figure 1 depicts Bland–Altman plots and scatter plots for gait speed collected from the chest device (with SKDH-gait) versus gait mat and versus APDM 6-sensor set, during in-lab walking tasks at normal self-paced speed. Results for other gait endpoints, reference devices, and self-paced walking speeds are reported in the Supplementary Material.

**Table 2:** Bias with LoA, mean absolute difference, mean percent error, ICC with LB and UB, and Pearson’s R with *p*-value computed between gait speed collected from the chest device (with SKDH-gait) versus the instrumented gait mat (first row) and versus the APDM 6-sensor set (second row), during in-lab walking tasks at different self-paced speeds, in-lab simulated activities, and outside-lab activities.

Reference device	Task	Bias (LoA) (m/s)	MAD (m/s)	MPE (%)	ICC (LB, UB)	Pearson’s R (*p*-value)
Gait mat	In-lab walking at slow speed	− 0.127 (− 0.242, − 0.013)	0.137	− 21.70	0.627 (− 0.073, 0.896)	0.919 (*P* < .001)
	In-lab walking at normal speed	− 0.162 (− 0.336, 0.012)	0.162	− 16.03	0.501 (− 0.095, 0.831)	0.813 (*P* < .001)
	In-lab walking at fast speed	− 0.209 (− 0.533, 0.115)	0.210	− 15.74	0.430 (− 0.102, 0.763)	0.663 (*P* < .001)
APDM 6-sensor set	In-lab walking at slow speed	− 0.027 (− 0.153, 0.098)	0.053	− 3.824	0.882 (0.716, 0.952)	0.917 (*P* < .001)
	In-lab walking at normal speed	− 0 − 0.228, 0.099)	0.077	− 6.589	0.745 (0.287, 0.905)	0.819 (*P* < .001)
	In-lab walking at fast speed	− 0.099 (− 0.393, 0.195)	0.134	− 7.756	0.611 (0.181, 0.834)	0.686 (*P* < .001)
	In-lab simulated activities	0.055 (− 0.103, 0.212)	0.079	4.381	0.734 (0.329, 0.896)	0.822 (*P* < .001)
	Outside-lab activities	− 0.027 (− 0.105, 0.160)	0.056	2.191	0.898 (0.755, 0.959)	0.908 (*P* < .001)

Error metrics are computed as difference between gait speed collected from the chest device and that collected from the reference device.

*LoA* Limits of Agreement, *MAD* Mean absolute difference, *MPE* Mean percent error, *ICC* intra-class correlation coefficient, *LB* Lower bound, *UB* Upper bound.

Compared to the gait mat, gait speed computed by SKDH-gait run on the chest device shows a negative bias, consistent across the measurement range, which increases for a higher perceived walking speed (SKDH-gait chest—Gait mat gait speed (m/s) = − 0.127 (− 20.18%), − 0.162 (− 16.04%), − 0.209 (− 15.74%) for the slow, normal, fast self-paced walking speeds, respectively). ICC and Pearson’s R show a moderate agreement and correlation, which increase for a slower perceived walking speed. Minimum values of ICC = 0.430 and R = 0.663 are observed during the walking task at fast self-paced speed.

Compared to the APDM 6-sensor set, gait speed estimated by SKDH-gait shows lower mean bias compared to gait mat, and exhibits a good-to-excellent agreement (ICC = 0.882, 0.745, 0.611, and R = 0.917, 0.819, 0.686, for slow, normal, and fast self-paced walking speed, respectively). The mean bias exhibits a limited trend throughout the measurement range only for the slow self-paced walking speed, as visible in the Supplementary Fig. S1.

Cadence computed by SKDH-gait run on the chest device shows a consistent bias throughout the measurement range compared to the gait mat (maximum bias value of 3.801 (3.031%) steps/min for the fast self-paced walking speed). ICC shows excellent agreement, regardless of the walking speed (ICC > 0.792), and a strong correlation (R > 0.848) is observed for all walking speeds.

Compared to APDM 6-sensor set, SKDH-gait run on the chest device underestimates cadence during in-lab walking tasks, with a bias which increases with the self-paced walking speed: (SKDH-gait chest—APDM 6-sensor set cadence (steps/min) = − 0.312 (− 0.370%), − 1.749 (− 1.657%), − 2.694 (− 2.197%) for slow, normal, and fast walking speeds, respectively). ICC reflects an excellent agreement between cadence from chest device and APDM 6-sensor set, regardless of the self-paced walking speed (ICC > 0.94). Similarly, Pearson’s R shows a strong correlation for all the self-paced walking speeds (R > 0.98).

Excellent agreement and strong correlations were observed for stride time regardless of the self-paced walking speed, both for the comparison against the gait mat (ICC > 0.966 and R > 0.974) and against APDM 6-sensor set (ICC > 0.951 and R > 0.987). Bias is limited regardless of the walking speed and, as visible in the Bland–Altman plots (reported in the Supplementary Figs. S1–S3), it is constant throughout the measuring range, for both the reference devices.

The agreement between stride length from SKDH-gait run on the chest device and the two reference devices is the lowest among the gait endpoints under analysis. When compared to the gait mat, stride length is, on average, underestimated, and the mean absolute difference is approximately 0.16 m for all walking speeds. ICC shows a poor-to-moderate agreement, while Pearson’s R still expresses strong correlation (R > 0.708 for all walking speeds). When compared to the APDM 6-sensor set, a lower mean absolute difference is observed (maximum value of 0.101 m observed in the walking task at fast self-paced speed), and the ICC suggests good agreement (ICC > 0.683 for all walking speeds). In line with literature results^28^, as the self-paced speed increased, the mean percent error decreased (from − 19.22% at slow self-paced speed to -14.59% at fast self-paced speed).

Bland–Altman plots (reported in the Supplementary Figs. S1–S3) show that the bias has no remarkable trend throughout the measuring range, for cadence, stride time, and stride length, for both the reference devices.

### Comparison of gait endpoints across multiple devices and in-lab walking tasks

The effect of device and self-paced walking speed on the agreement between gait endpoints is evaluated via mixed model regression. For each gait endpoint, the *p*-values of the ANOVA testing the overall effect of the device, and the *p*-values of the post-hoc tests comparing chest and reference devices are reported in Table 3, together with the estimates (and their 95% confidence intervals) of the least square mean differences between chest and reference devices, for different self-paced speed.

**Table 3:** Comparison between gait endpoints from the chest device (SKDH-gait) and reference devices for in-lab walking tasks at self-paced speeds. For each gait endpoint, the *p*-value of the ANOVA test to assess the overall effect of the device is reported, together with the *p*-value of the post-hoc test assessing the difference between chest and reference devices. The estimates and 95% confidence interval of the least square mean differences between chest and reference devices are reported for each self-paced speed. An overall device effect is observed for all endpoints but stride time. The chest device is significantly different from the gait mat and APDM 6-sensor set in terms of gait speed and stride length. No significant difference is observed between chest and lumbar devices.

Reference devices	Self-paced walking speed	Endpoint (overall effect of device)			
		Gait speed (*P* < .001)	Cadence (*P* = .02)	Stride time (*P* = .84)	Stride length (*P* < .001)
Gait mat		***P* < .001**	*P* = .19	*P* = .49	***P* < .001**
	Slow	− 0.127 [− 0.204, − 0.051]	0.508 [− 3.678, 4.693]	0.014 [− 0.051, 0.079]	− 0.176 [− 0.229, − 0.122]
	Normal	− 0.162 [− 0.238, − 0.086]	0.563 [− 3.623, 4.749]	0.009 [− 0.056, 0.074]	0.165 [− 0.219, − 0.112]
	Fast	− 0.209 [− 0.285, − 0.133]	3.801 [− 0.385, 7.987]	0.017 [− 0.048, 0.082]	− 0.162 [− 0.215, − 0.108]
APDM 6-sensor set		***P* = .005**	*P* = .20	*P* = .53	***P* = .009**
	Slow	− 0.027 [− 0.103, 0.049]	− 0.312 [− 4.498, 3.873]	0.000 [− 0.065,0.065]	− 0.028 [− 0.081, 0.025]
	Normal	− 0.064 [− 0.140, 0.012]	− 1.749 [− 5.935, 2.437]	− 0.014 [− 0.051, 0.080]	− 0.050 [− 0.103, 0.004]
	Fast	− 0.099 [− 0.175, − 0.023]	− 2.694 [− 6.880, 1.491]	0.022 [− 0.043, 0.087]	− 0.045 [− 0.099, 0.008]
SKDH-gait lumbar		*P* = .66	*P* = .88	*P* = .97	*P* = 0.79
	Slow	0.011 [− 0.065, 0.087]	− 0.876 [− 5.062, 3.310]	0.011 [− 0.054, 0.076]	0.025 [− 0.028, 0.079]
	Normal	− 0.032 [− 0.108, 0.044]	0.410 [− 3.776, 4.596]	− 0.005 [− 0.070, 0.060]	− 0.035 [− 0.088, 0.018]
	Fast	− 0.009 [− 0.085, 0.067]	1.026 [− 3.160, 5.211]	− 0.004 [− 0.069, 0.061]	− 0.003 [− 0.056, 0.051]

Significant values are in [bold].

**Figure 2:**
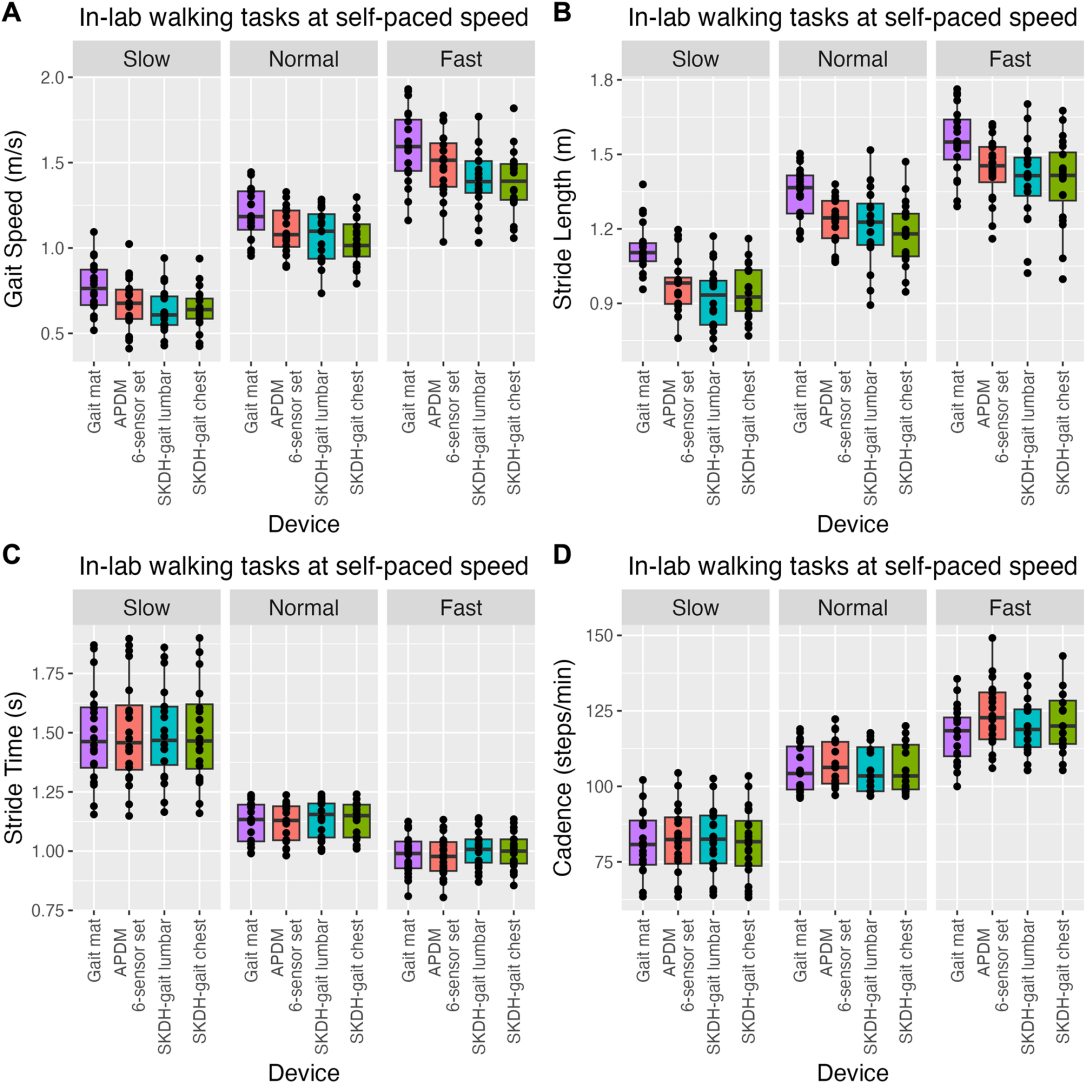
Gait parameters across devices during the walking task at normal speed. Distributions of gait speed (panel **A**), stride length (panel **B**), stride time (panel **C**), and cadence (panel **D**) from the gait mat (magenta), APDM 6-sensor set (red), lumbar device with SKDH-gait (cyan), and chest device with SKDH-gait (green), for in-lab walking tasks at self-paced speed. Data are reported in boxplot representation: the central horizontal lines represent the median, the boxes mark the interquartile range, black lines are the whiskers, and the black dots indicate each individual data value.

Figure 2 shows the distribution of gait endpoints collected by different devices during the in-lab walking tasks at slow, normal, and fast self-paced speed. We observe consistent distributions of devices and endpoints across different walking speeds: gait speed, stride length, and cadence increase for fast walk compared to slow walk, while stride time decreases towards higher self-paced speeds.

Compared to the gait mat, all other devices show shorter stride length, especially in the slow walking task. Since gait speed is given by the ratio between stride length and stride time^31^, similar trends across devices are observed for the distributions of stride length and gait speed.

The ANOVA test shows a significant effect of the self-paced walking speed for all the endpoints (*P* < 0.001), suggesting that all gait endpoints differentiate between the three different self-paced walking speeds, as expected. In addition, as already observed in the literature^13^, the ANOVA test shows a significant effect of the device for all endpoints but stride time (*P* < 0.001 for gait speed and stride length, *P* = 0.02 for cadence, *P* = 0.84 for stride time), suggesting that stride time is not significantly different across devices. Interestingly, the interaction between device and self-paced walking speed is not significant (*P* > 0.50) for all gait endpoints, showing that the error between devices is not affected by the self-paced walking speed.

Post-hoc tests for the difference between devices show no significant difference between chest and lumbar devices for any of the endpoints, demonstrating that the performance of SKDH-gait remains the same with respect to the position of the device.

In addition, gait speed and stride length are significantly different between chest device and both the gait mat and APDM 6-sensor set. Both parameters are underestimated by SKDH-gait applied to the chest device, but the bias is homogenous, in corroboration with the previous findings for SKDH-gait applied to the lumbar device^13^.

Finally, while the ANOVA test shows a significant effect of the device for cadence, post-hoc tests show that this effect is not driven by the chest device, which is not significantly different from reference devices; instead, there is a significant difference in cadence between APDM 6-sensor set and the gait mat. Notably, in this analysis, a correction for multiple comparisons would have led to a different conclusion for those *p*-values close to 0.05, including that related to the overall effect of the device for cadence.

### Agreement of gait endpoints derived from chest device and reference devices during simulated in-lab and outside-lab activities

In this section, the agreement between gait parameters collected with SKDH-gait run on the chest device, and those collected using APDM 6-sensor set during the in-lab simulated activities and outside-lab free-living activities is presented.

**Figure 3:**
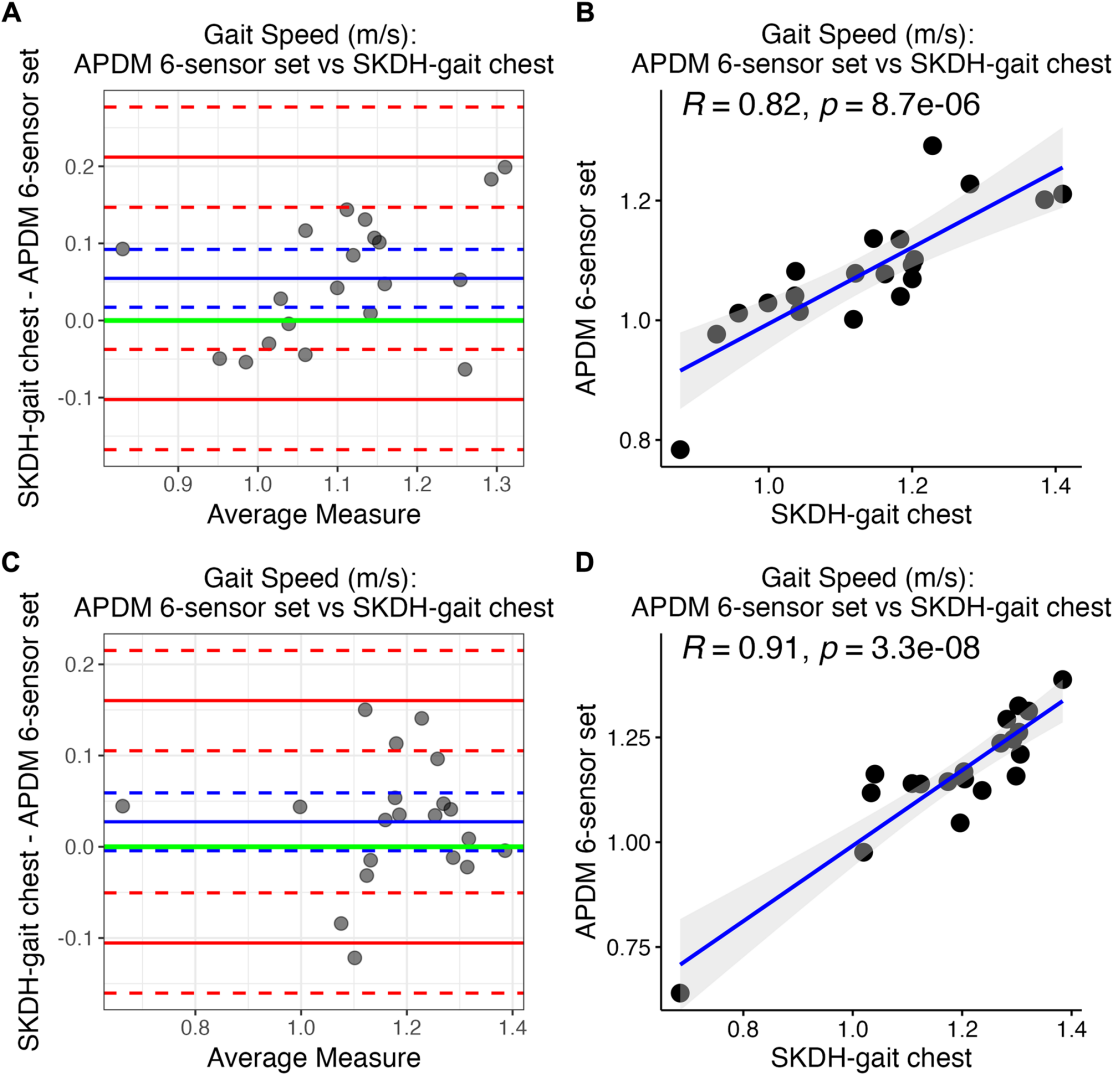
Assessment of gait speed during activity tasks. Assessment of gait speed estimated from the chest device (SKDH-gait) against the APDM 6-sensor set, during in-lab simulated activities (panels **A**, **B**), and outside-lab activities (panels **C**, **D**). Bland–Altman plots (panels **A**, **C**) show acceptable bias (blue solid line) and narrow LoA (red solid lines), for both the environments. Corresponding confidence intervals are in dashed lines, while green solid line represents the 0-threshold. Scatter plots (panels **B**, **D**) show highly correlated measurements for both the environments. Regression line (solid blue line) with 95% confidence interval (shaded gray area) are depicted, and the Pearson’s R with the related *p*-value are annotated in the figure.

Error metrics, Pearson’s R, and ICC are reported in the last 2 rows of Table 2, for gait speed only. Figure 3 depicts Bland–Altman plots and scatter plots for gait speed collected from chest device (with SKDH-gait) versus APDM 6-sensor set, during in-lab simulated activities, and outside-lab activities. Results for other gait endpoints, and reference devices are reported in the Supplementary Material.

Gait parameters estimated by SKDH-gait run on the chest device show a good-to-excellent agreement against the parameters estimated by APDM 6-sensor set, during activities performed both in-lab and outside-lab. ICC is < 0.75 only for stride length (ICC = 0.701) and gait speed (ICC = 0.734), for the in-lab simulated activities. Compared to the in-lab simulated activities, in the outside-lab activities the difference between the chest device and APDM 6-sensor set is lower, with narrower LoA for all endpoints but 95th percentile of gait speed (for this endpoint, the mean percent error is 1.274% for in-lab activities, and 4.779% for outside-lab activities). More precisely, for all endpoints collected during the outside-lab activities, the 0-threshold (green line in the Bland–Altman plots reported in Fig. 3C, and Supplementary Fig. S5) falls within the 95% confidence interval of the bias between chest device and APDM 6-sensor set, demonstrating that the bias is not significantly different than 0. In addition, ICC and correlation between gait endpoints collected from chest device and APDM 6-sensor set are higher during the outside-lab activities, compared to the in-lab simulated activities. This suggests that gait metrics derived from a single chest device provide accurate and reliable gait characterization during longer walking activities, simulating free-living environment.

Nonetheless, a strong Pearson’s correlation between chest device and APDM 6-sensor set is observed for all the endpoints: both in-lab and outside-lab, the lowest correlation is observed in stride length (R: 0.786 for in-lab simulated activities, and R: 0.841 for outside-lab activities), in line with the results observed during the in-lab walking tasks.

**Figure 4:**
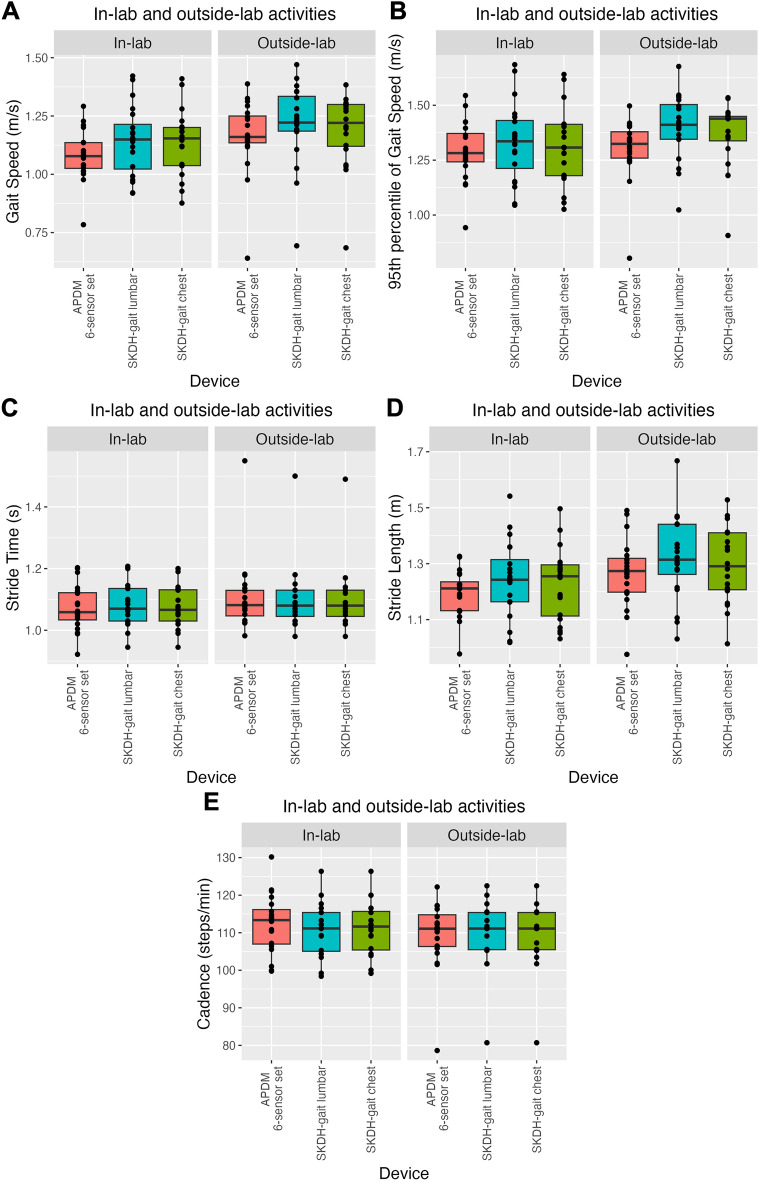
Gait parameters across devices during activity tasks. Distributions of gait speed (panel **A**), 95th percentile of gait speed (panel **B**), stride time (panel **C**), stride length (panel **D**), and cadence (panel **E**), from APDM 6-sensor set (red), lumbar device with SKDH-gait (cyan), and chest device with SKDH-gait (green), for in-lab and outside-lab activities. Data are reported in boxplot representation: the central horizontal lines represent the median, the boxes mark the interquartile range, black lines are the whiskers, and the black dots indicate outliers.

Figure 4 shows the distribution of gait endpoints collected by different devices during in-lab and outside-lab activities. The distributions highly overlap for all endpoints, devices, and tasks.

The effect of device and environment (i.e., in-lab or outside-lab) on the agreement between different endpoints is evaluated via mixed model regression. ANOVA test shows a significant effect of the environment for gait speed (*P* = 0.02), cadence (*P* = 0.04), and stride length (P = 0.008), which were higher during the outside-lab activities, compared to the in-lab simulated activities. No significant effect of device is observed for any endpoints. Finally, there is no significant effect of the interaction between device and environment. Given the above-mentioned considerations, no post-hoc tests are performed for pairwise comparisons.

### Comparison between chest and lumbar devices in terms of SKDH-gait-detected walking bouts during outside-lab activities

To reduce the dependency from the bout outliers, bouts shorter than 9 s or having less than 4 gait cycles collected during the outside-lab activities were discarded for this analysis, resulting in the removal of 448 bouts (37%) from the chest device, and 294 bouts from the lumbar device (36%).

**Figure 5:**
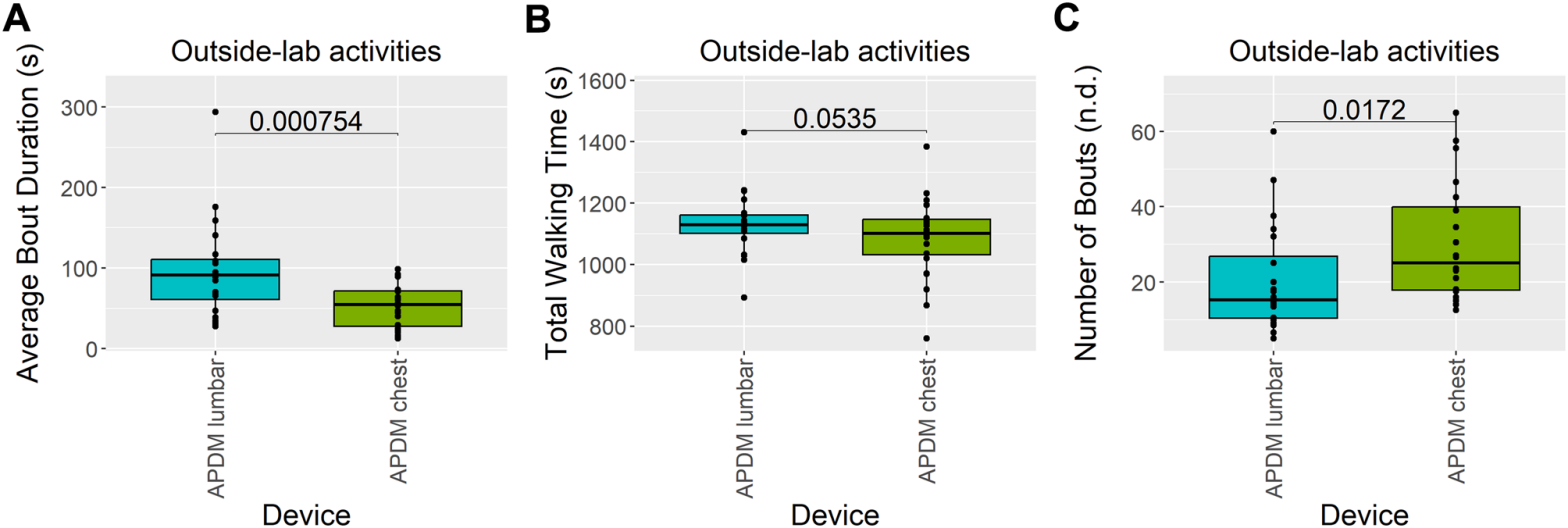
Comparison of bout parameters from chest and lumbar devices. Distributions of average bout duration (panel **A**), total walking time (panel **B**), number of bouts (panel **C**), and total number of steps (panel **D**) from SKDH-gait applied to lumbar (cyan) and chest (green) devices during outside-lab activities. Data are reported in boxplot representation: the central horizontal lines represent the median, the boxes mark the interquartile range, black lines are the whiskers, and the black dots indicate outliers. The *p*-value of a pairwise t-test is also depicted.

Figure 5 depicts the average bout duration, the total walking time, the number of bouts, and the total number of steps derived by SKDH-gait when run on the lumbar device (cyan) and on the chest device (green), during the outside-lab activities, averaged across visits. *P*-values of the paired t-test to assess their mean difference are also reported.

SKDH-gait run on the chest device significantly underestimates the average bout duration, and significantly overestimates the number of bouts, compared to the lumbar device. However, the total walking time, as well as the total number of steps are not significantly different between the two devices (*P* = 0.0885 and *P* = 0.0811, respectively). This analysis suggests that when run on a chest device, SKDH-gait might detect a long walking bout as multiple consecutive shorter walking bouts, still estimating the total walking time and the total number of steps comparable to the total walking time and total number of steps detected by the lumbar device.

## Discussion

In this work, we assessed the validity of gait speed (and other gait parameters, such as cadence, stride time, and stride length) derived using an in-house built open source algorithm, namely SKDH-gait^31^, applied to a chest accelerometer (single APDM device), in comparison to different reference devices (APDM 6-sensor set, GAITRite gait mat, and single APDM lumbar accelerometer), during various in-lab and outside-lab tasks. The results presented in this work demonstrated that SKDH-gait run on a single chest-worn accelerometer can accurately track gait during both controlled and naturalistic environments. Specifically, the chest and lumbar devices showed no significant difference and excellent agreement for all SKDH-derived gait endpoints and in-lab/outside-lab tasks, suggesting that the reliability of SKDH-gait is not affected by the accelerometer’s body location.

During in-lab walking tasks at slow, normal, and fast self-paced speed, gait speed from the chest accelerometer showed moderate agreement in comparison to the gait mat, and a good-to-excellent agreement and high correlation in comparison to the APDM 6-sensor set. Compared to both the reference devices, the chest accelerometer underestimated stride length (maximum bias against the gait mat of − 0.178 m in the walking task for fast self-paced speed) and gait speed (maximum bias against the gait mat of − 0.209 m/s in the walking task for fast self-paced speed), but importantly the bias was systematic, in line with previous findings^13^. For stride time and cadence there were no significant differences between the chest device and reference devices. These results suggest the effectiveness of SKDH-gait run on a chest accelerometer to measure gait during in-lab walking tasks.

During in-lab and outside-lab activities, mimicking activities of daily living, all gait endpoints from the chest accelerometer showed a good-to-excellent agreement in comparison to APDM 6-sensor set, with a slightly higher bias in the in-lab simulated activities (for gait speed, bias of 0.055 m/s in the in-lab simulated activities, and -0.027 in the outside-lab activities). Furthermore, we assessed the reliability of SKDH-gait run on the chest accelerometer to capture the step count and the total walking time: when compared to the results obtained from the lumbar-worn accelerometer, it tends to fragment longer bouts in multiple shorter bouts, resulting in underestimation of the average bout duration and overestimation of the number of bouts, but without effecting the total walking time and step counts. Finally, the test–retest reliability analysis (reported in the Supplementary Material) showed that SKDH-gait run on the chest accelerometer can reliably reproduce the results over time. These results provide evidence on the effectiveness of SKDH-gait run on a chest accelerometer to measure gait during activities of daily living or free-living environments. Additionally, both the chest- and lumbar-worn devices deployed in this study are based on a single sensor, thus they are ergonomically and economically more advantageous than multiple sensors sets.

These results are particularly promising for gait assessment: while traditional clinical research tools, employed during episodic in-lab or in-clinic visits, might not provide sufficient objectivity and repeatability, the benefit of using DHTs, such as wearable accelerometers, to continuously and passively measure gait in free-living environment is increasingly being recognized by the scientific community, pharmaceutical industry, and patients^36^. The choice of a wearable accelerometer’s body location covers an important role for its deployment in clinical trials, especially for long-term monitoring, as it affects participants’ comfort (and, in turn, their compliance), as well as data type and quality. For example, a chest patch is certainly preferred to measure ECG and other vital signs, a smartwatch is often used to monitor acceleration and track physical activity levels and sleep patterns, and a lumbar belt mounted with movement sensors is commonly deployed to derive gait parameters. Notably, measuring the above-mentioned bio-signals all together would be beneficial, but wearing two or more wearable devices simultaneously might be cumbersome for participants, e.g., charging or taking on and off multiple devices. The use of separate sensors can also risk data quality, especially for multivariate analyses, as it might require additional processing pipelines to synchronize and match data from multiple sources. This work demonstrates that a chest accelerometer can accurately characterize gait, thus supporting the deployment of a single multimodal chest-worn device to simultaneously measure both gait parameters and other vital signs, such as heart rate or respiratory rate, typically captured from a chest location. Furthermore, a chest location can potentially offer more reliability in the estimation of other parameters, such as sway, gait stability, and risk of fall. This will have the potential of improving users’ experience^37^, resulting in a more comprehensive and holistic view of individuals’ health and well-being, and providing a more complete picture of their physiological and physical conditions^38,39^.

When combined with machine learning/artificial intelligence tools, multimodal DHTs could also lead to more robust decision support systems to assist clinicians with diagnosis, prognosis, and personalized treatments of specific diseases^40^. Several published works stress the importance of gathering vital signs and accelerometry data simultaneously. For example, Park et al.^41^ suggested that the prediction of stroke during daily activities, such as driving, sleeping, and working, requires the integration of multiple bio-signals, including gait, ECG, and respiratory rate. Pandey et al.^42^ recognized that the simultaneous monitoring of gait speed and heart rate could provide better understanding of the association between frailty and heart failure in older adults, in turn improving their functional status, quality of life, and long-term clinical outcomes. In a more recent study, separate DHTs to measure gait and heart rate were deployed in a frail cohort, showing a significant effect of the frailty phenotype on the association between heart rate (HR) dynamics and walking performance^43^. Finally, the LINK-HF study demonstrates the effectiveness of a multi-modal chest device to measure acceleration and vital signs in combination with a personalized machine learning platform to accurately predict rehospitalization for heart failure^44^. Along with these lines, our work opens to the possibility to collect gait and vital signs simultaneously, by deploying a single multimodal chest-worn device.

Given the small sample size of this study, further validation in a larger population is required and recommended. Similarly, clinical validation in a more representative population should be performed, including children, the elderly, and patients with underlying cardiopulmonary conditions. Of note, in this study we only focused on a chest accelerometer positioned on the sternum, further studies can evaluate whether placement superior (e.g., suprasternal notch), or lateral, or even inferior (e.g., umbilicus) to the sternum would result in similar findings. The chest accelerometer deployed in this study was mounted on a chest harness, worn on the top of the clothes; further analyses are needed to evaluate the accuracy of typical chest patches, which are adhered to the skin^45,46^ and, as such, might be more stable when worn on the body, potentially leading to even more accurate gait tracking, and longer wear time.

In conclusion, while this work will inform the accuracy of future studies using a single chest accelerometer for free-living gait analysis, our results also demonstrate the reliability of measuring gait from a chest location, encouraging the implementation of accelerometers as another modality in those chest-worn devices for vital signs monitoring.

### Supplementary Information

Supplementary Information.

## Data Availability

The data that support the findings of this study are available from Pfizer but restrictions apply to the availability of these data, which were used under license for the current study, and so are not publicly available. Data are however available from the corresponding author upon reasonable request and with permission of Pfizer.
