# Supplementary material for: Measuring gait parameters from a single chest-worn accelerometer in healthy individuals: a validation study
Source: Sci Rep. 2024 Jun 17;14:13897. doi: 10.1038/s41598-024-62330-6 (PMC11183133; doi:10.1038/s41598-024-62330-6)
Supplement: Supplementary file 1 — Supplementary Information. [file 41598_2024_62330_MOESM1_ESM.docx]

## **Measuring gait parameters from a single chest-worn accelerometer in healthy individuals: a validation study**

## N Camerlingo^1^, PhD, X Cai^1^, MD, L Adamowicz^1^, MSc, M Welbourn^1^, BSc, DJ Psaltos^1^, MSc, H Zhang^1^, PhD, A Messere^1^, BSc, J Selig^1^, BA, W Lin^1^, PhD, P Sheriff^1^, BSc, C Demanuele^1^, PhD, M Santamaria^1^, MD, FI Karahanoglu^1^, PhD

## ^1^Pfizer, Inc., Cambridge, MA, USA

#### Supplementary Material


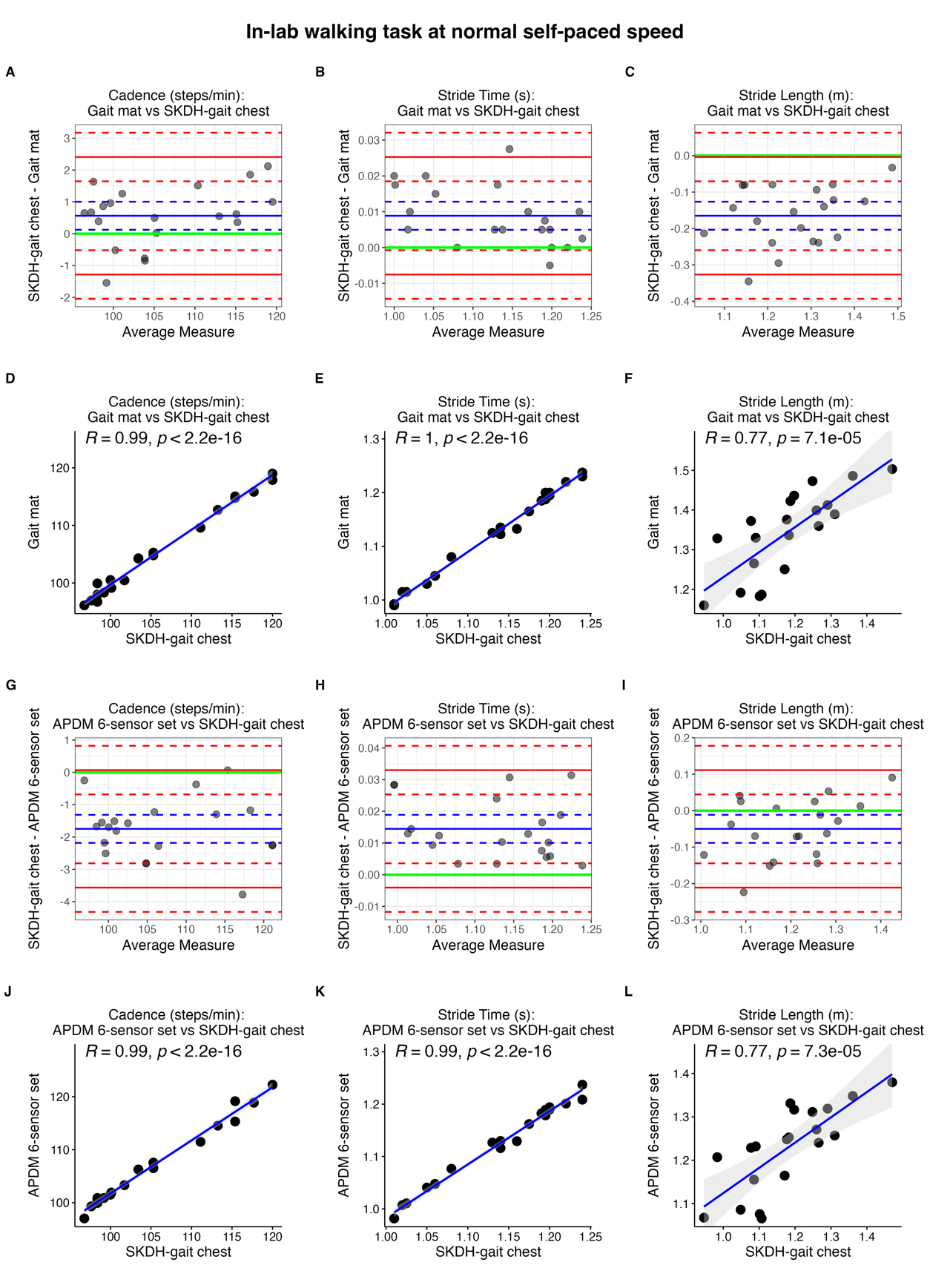


***Supplementary Figure S1.*** *Assessment of gait parameters estimated from the chest accelerometer (with SKDH-gait) against an instrumented gait mat (panels A to F), and the APDM 6-sensor set (panels G to L), during in-lab walking tasks at normal self-paced speed: cadence (column 1), stride time (column 2), and stride length (column 3).*


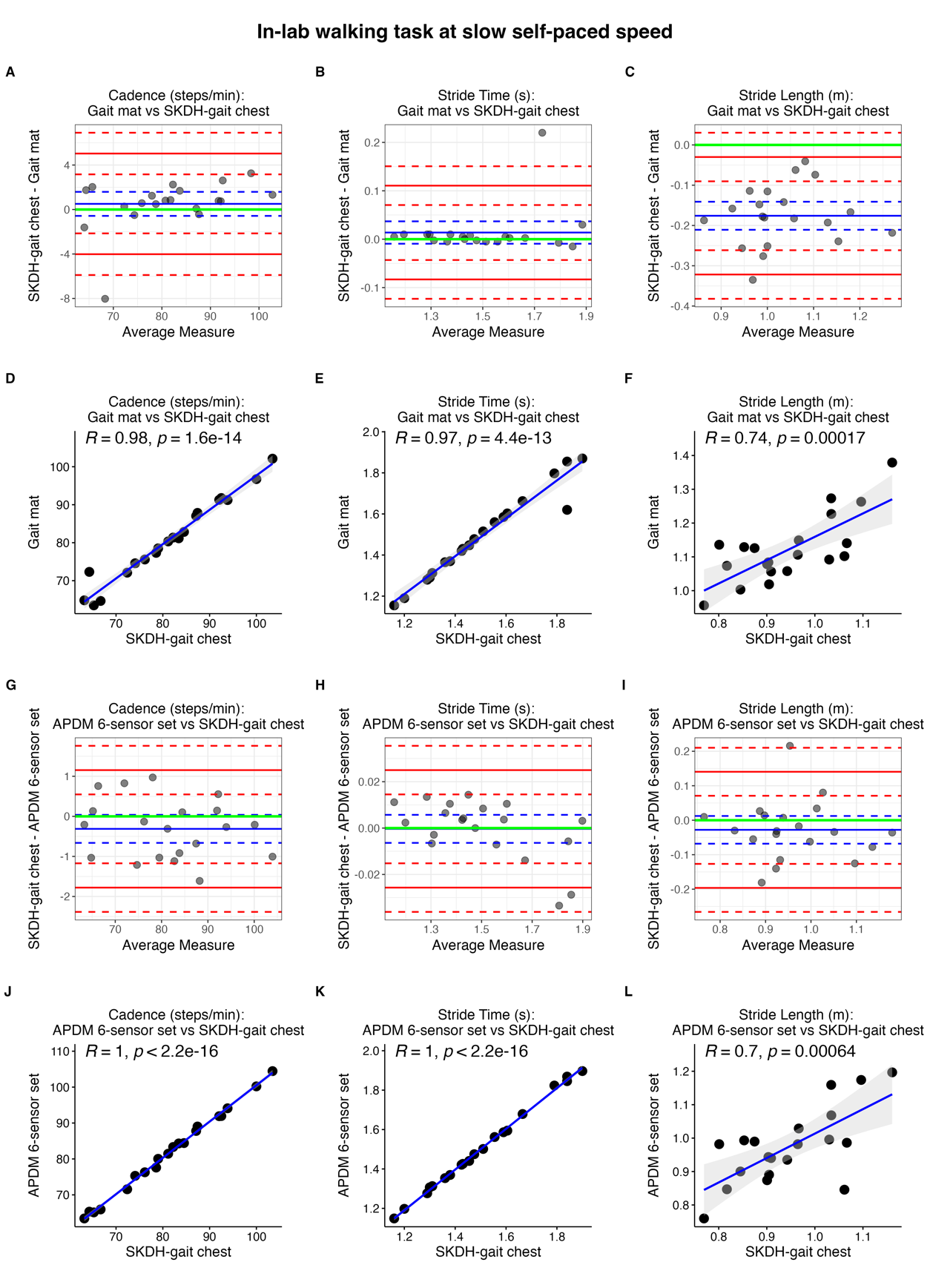


***Supplementary Figure S2.*** *Assessment of gait parameters estimated from the chest accelerometer (with SKDH-gait) against an instrumented gait mat (panels A to F), and the APDM 6-sensor set (panels G to L), during in-lab walking tasks at slow self-paced speed: cadence (column 1), stride time (column 2), and stride length (column 3).*


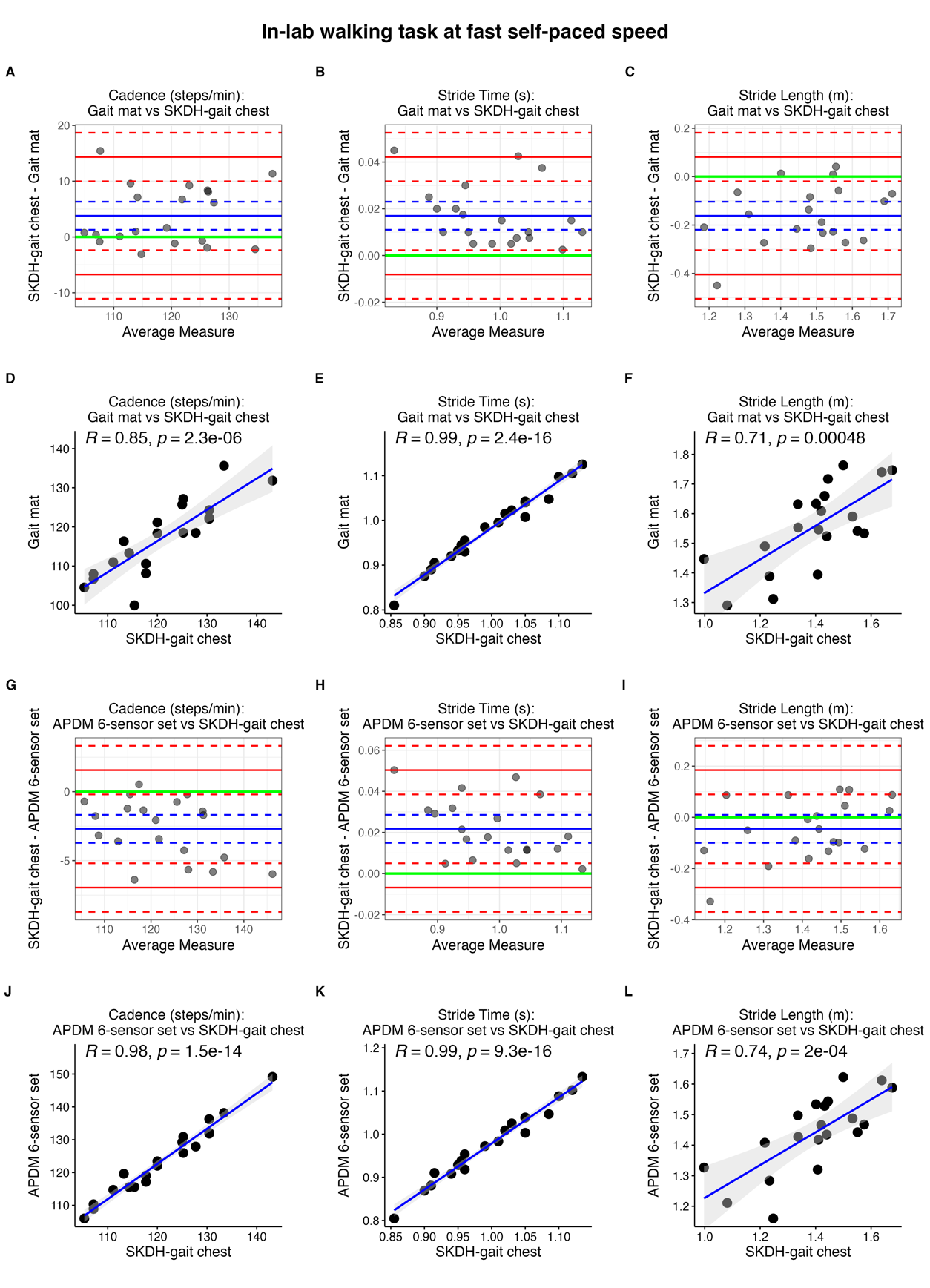


***Supplementary Figure S3.*** *Assessment of gait parameters estimated from the chest accelerometer (with SKDH-gait) against an instrumented gait mat (panels A to F), and the APDM 6-sensor set (panels G to L), during in-lab walking tasks at fast self-paced speed: cadence (column 1), stride time (column 2), and stride length (column 3).*


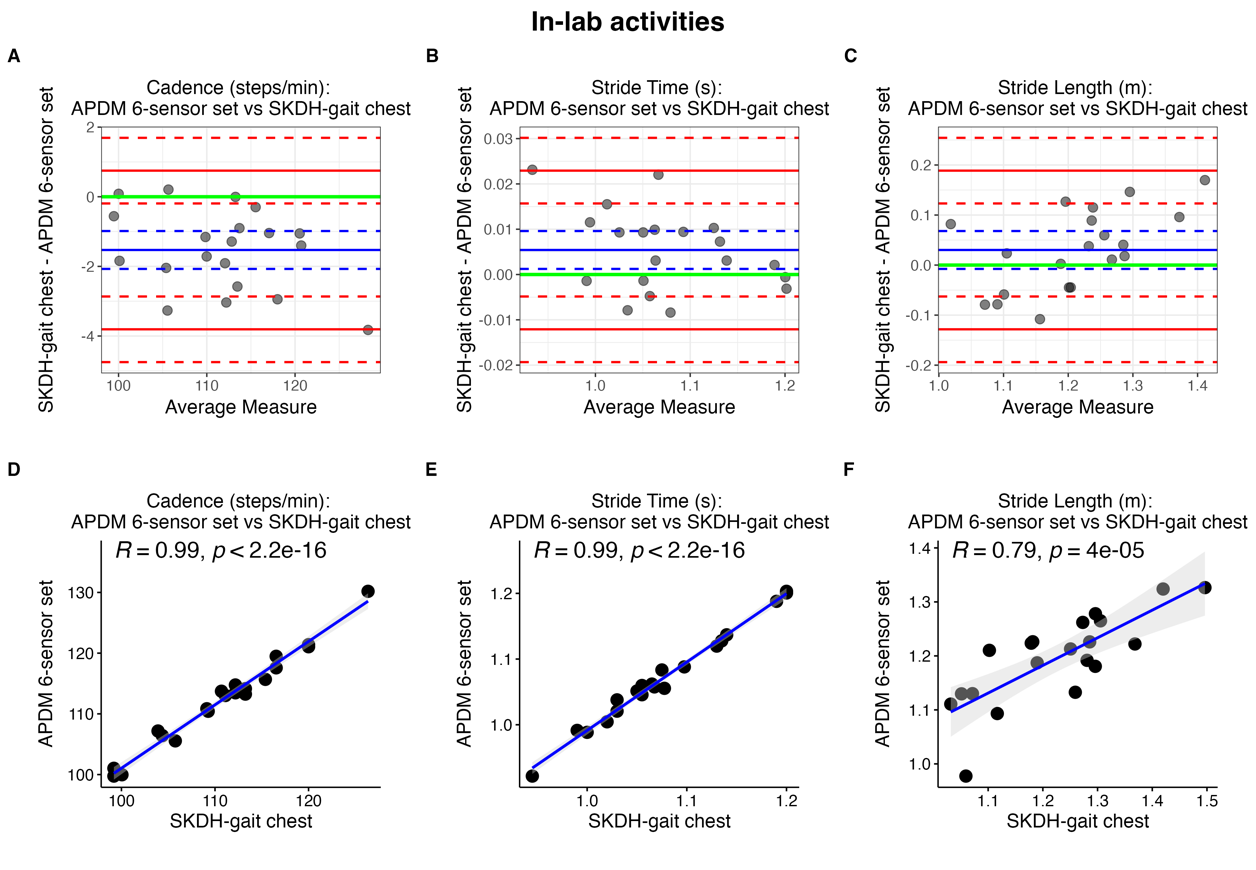


***Supplementary Figure S4.*** *Assessment of gait parameters estimated from the chest accelerometer (SKDH-gait) against the APDM 6-sensor set, during in-lab simulated activities: cadence (column 1), stride time (column 2), and stride length (column 3).*


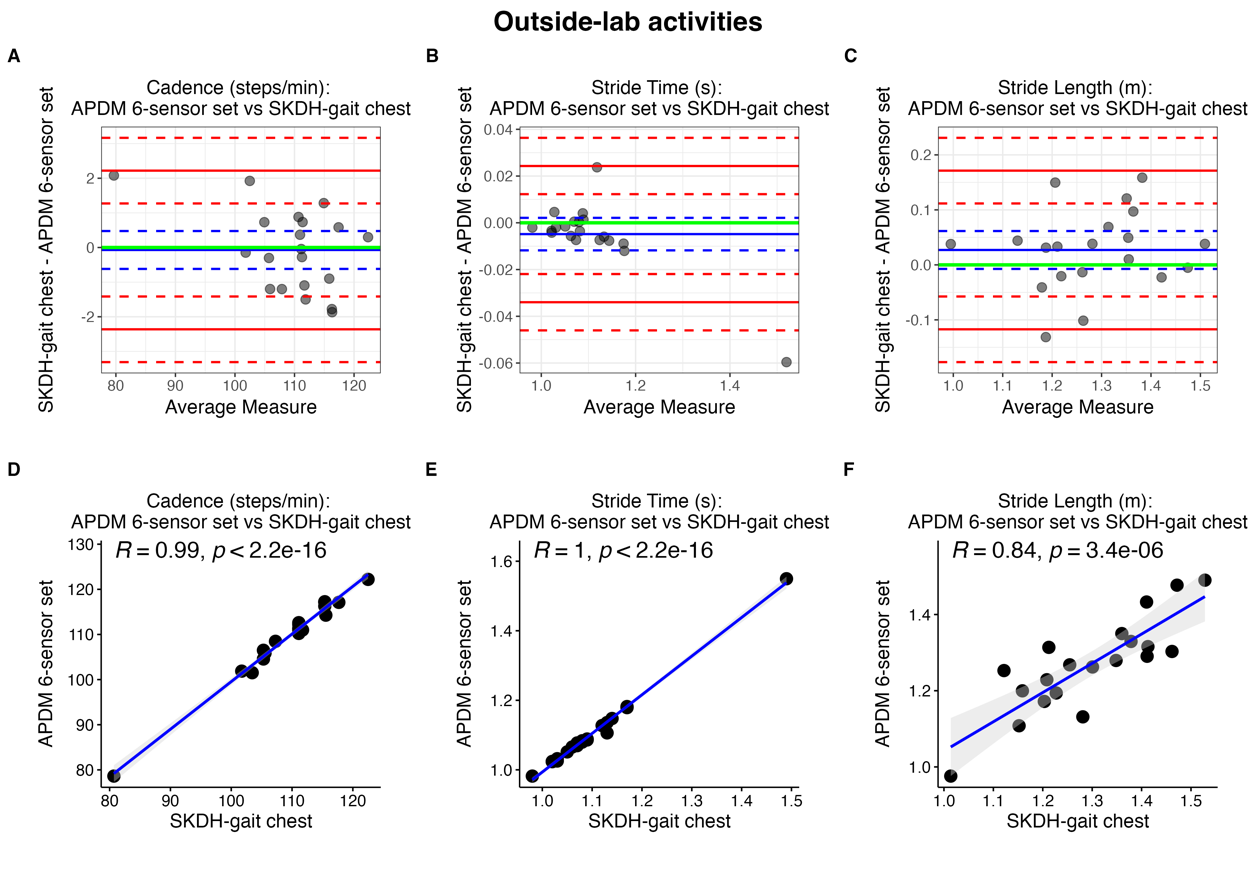


***Supplementary Figure S5.*** *Assessment of gait parameters estimated from the chest accelerometer (SKDH-gait) against the APDM 6-sensor set, during outside-lab activities: cadence (column 1), stride time (column 2), and stride length (column 3).*

| ***Supplementary Table S1:*** *Bias with LoA, mean absolute difference, mean percent error, ICC with LB and UB, and Pearson’s R with p-value computed between gait endpoints (column 3: cadence, stride time, stride length, and gait speed) collected from the chest device (with SKDH-gait) vs different reference devices (column 1: the instrumented gait mat, the APDM 6-sensor set, and the lumbar accelerometer with SKDH-gait), during different tasks (column 2: in-lab walking at slow, normal, and fast self-paced speed, in-lab simulated activities, and outside-lab activities). Error metrics are computed as difference between gait endpoint collected from the chest device and that collected from the reference devices. (LoA: Limits of Agreement, MAD: Mean absolute difference, MPE: Mean percent error, ICC: intra-class correlation coefficient, LB: Lower bound, UB: Upper bound)* | | | | | | | |
| --- | --- | --- | --- | --- | --- | --- | --- |
| **Reference device** | **Task** | **Endpoint** | **Bias (LoA)** | **MAD** | **MPE, %** | **ICC (LB, UB)** | **Pearson’s R (p-value)** |
| **Gait mat** | **In-lab walking at slow speed** | **Cadence** | 0.508  (-4.018, 5.033) | 1.565 | 0.430 | 0.979  (0.948, 0.991) | 0.982 (*P*<.001) |
|  |  | **Stride time** | 0.014  (-0.083, 0.111) | 0.018 | 0.797 | 0.972  (0.932, 0.989) | 0.974 (*P*<.001) |
|  |  | **Stride length** | -0.176  (-0.322, -0.030) | 0.176 | -19.22 | 0.306  (-0.071, 0.698) | 0.745 (*P*<.001) |
|  |  | **Gait speed** | -0.127  (-0.242, -0.013) | 0.127 | -20.18 | 0.627  (-0.073, 0.896) | 0.919  (*P*<.001) |
|  | **In-lab walking at normal speed** | **Cadence** | 0.563  (-1.283, 2.409) | 0.932 | 0.509 | 0.991  (0.969, 0.997) | 0.994 (*P*<.001) |
|  |  | **Stride time** | 0.009  (-0.008, 0.025) | 0.009 | 0.810 | 0.989  (0.884, 0.997) | 0.996 (*P*<.001) |
|  |  | **Stride length** | -0.165  (-0.326, -0.004) | 0.165 | -14.59 | 0.383  (-0.088, 0.758) | 0.770 (*P*<.001) |
|  |  | **Gait speed** | -0.162 (-0.336, 0.012) | 0.162 | -16.04 | 0.501  (-0.095, 0.831) | 0.813  (*P*<.001) |
|  | **In-lab walking at fast speed** | **Cadence** | 3.801  (-6.729, 14.33) | 4.790 | 3.031 | 0.792  (0.408, 0.923) | 0.848 (*P*<.001) |
|  |  | **Stride time** | 0.017  (-0.008, 0.042) | 0.017 | 1.741 | 0.966  (0.501, 0.992) | 0.989 (*P*<.001) |
|  |  | **Stride length** | -0.162  (-0.404, 0.081) | 0.168 | -12.52 | 0.457  (-0.102, 0.785) | 0.708 (*P*<.001) |
|  |  | **Gait speed** | -0.209  (-0.533, 0.115) | 0.210 | -15.74 | 0.430  (-0.102, 0.763) | 0.663  (*P*<.001) |
| **APDM 6-sensor set** | **In-lab walking at slow speed** | **Cadence** | -0.312 (-1.779, 1.154) | 0.661 | -0.370 | 0.998  (0.994, 0.999) | 0.998 (*P*<.001) |
|  |  | **Stride time** | 0.00 (-0.026, 0.025) | 0.010 | 0.040 | 0.998  (0.996, 0.999) | 0.999 (*P*<.001) |
|  |  | **Stride length** | -0.028 (-0.196, 0.141) | 0.067 | -3.320 | 0.685  (0.369, 0.861) | 0.697 (*P*<.001) |
|  |  | **Gait speed** | -0.027 (-0.153, 0.098) | 0.053 | -3.824 | 0.882  (0.716, 0.952) | 0.917  (*P*<.001) |
|  | **In-lab walking at normal speed** | **Cadence** | -1.749 (-3.565, 0.068) | 1.755 | -1.657 | 0.972  (0.229, 0.994) | 0.994 (*P*<.001) |
|  |  | **Stride time** | 0.014 (-0.004, 0.033) | 0.014 | 1.293 | 0.978  (0.484, 0.995) | 0.994 (*P*<.001) |
|  |  | **Stride length** | -0.050 (-0.211, 0.111) | 0.075 | -4.758 | 0.684  (0.298, 0.868) | 0.770 (*P*<.001) |
|  |  | **Gait speed** | -0.064 (-0.228, 0.099) | 0.077 | -6.589 | 0.745  (0.287, 0.905) | 0.819  (*P*<.001) |
|  | **In-lab walking at fast speed** | **Cadence** | -2.694 (-6.955, 1.566) | 2.747 | -2.197 | 0.948  (0.435, 0.987) | 0.982 (*P*<.001) |
|  |  | **Stride time** | 0.022 (-0.007, 0.050) | 0.022 | 2.231 | 0.951  (0.273, 0.989) | 0.987 (*P*<.001) |
|  |  | **Stride length** | -0.045 (-0.275, 0.185) | 0.101 | -4.074 | 0.683  (0.363, 0.860) | 0.739 (*P*<.001) |
|  |  | **Gait speed** | -0.099 (-0.393, 0.195) | 0.134 | -7.756 | 0.611  (0.181, 0.834) | 0.686  (*P*<.001) |
|  | **In-lab simulated activities** | **Cadence** | -1.529 (-3.808, 0.750) | 1.558 | -1.367 | 0.969  (0.528, 0.992) | 0.989 (*P*<.001) |
|  |  | **Stride time** | 0.005 (-0.012, 0.023) | 0.008 | 0.522 | 0.990  (0.965, 0.996) | 0.993 (*P*<.001) |
|  |  | **Stride length** | 0.030 (-0.128, 0.189) | 0.072 | 1.987 | 0.701  (0.392, 0.869) | 0.786 (*P*<.001) |
|  |  | **Gait speed** | 0.055 (-0.103, 0.212) | 0.079 | 4.381 | 0.734 (0.329, 0.896) | 0.822 (*P*<.001) |
|  |  | **95^th^ percentile of gait speed** | 0.023 (-0.163, 0.210) | 0.077 | 1.274 | 0.811 (0.589, 0.920) | 0.834 (*P*<.001) |
|  | **Outside-lab activities** | **Cadence** | -0.071 (-2.362, 2.219) | 0.959 | -0.025 | 0.992 (0.979, 0.997) | 0.993 (*P*<.001) |
|  |  | **Stride time** | -0.005 (-0.012, 0.023) | 0.008 | 0.522 | 0.991 (0.976, 0.996) | 0.997 (*P*<.001) |
|  |  | **Stride length** | 0.027 (-0.117, 0.171) | 0.061 | 1.866 | 0.826 (0.612, 0.927) | 0.841 (*P*<.001) |
|  |  | **Gait speed** | 0.027 (-0.105, 0.160) | 0.056 | 2.191 | 0.898 (0.755, 0.959) | 0.908 (*P*<.001) |
|  |  | **95^th^ percentile of gait speed** | 0.067 (-0.070, 0.204) | 0.083 | 4.779 | 0.803 (0.240, 0.937) | 0.885 (*P*<.001) |
| **SKDH-gait lumbar** | **In-lab walking at slow speed** | **Cadence** | -0.876  (-12.59, 10.83) | 1.970 | -1.536 | 0.863  (0.691, 0.943) | 0.861 (*P*<.001) |
|  |  | **Stride time** | 0.011  (-0.094, 0.115) | 0.021 | 0.519 | 0.967  (0.921, 0.987) | 0.971 (*P*<.001) |
|  |  | **Stride length** | 0.025  (-0.064, 0.115) | 0.043 | 2.783 | 0.905  (0.736, 0.964) | 0.933 (*P*<.001) |
|  |  | **Gait speed** | 0.011  (-0.071, 0.094) | 0.033 | 1.583 | 0.943  (0.863, 0.977) | 0.945 (*P*<.001) |
|  | **In-lab walking at normal speed** | **Cadence** | 0.410  (-1.409, 2.229) | 0.583 | 0.357 | 0.992  (0.979, 0.997) | 0.995 (*P*<.001) |
|  |  | **Stride time** | -0.005  (-0.023, 0.013) | 0.007 | -0.424 | 0.992  (0.976, 0.997) | 0.994 (*P*<.001) |
|  |  | **Stride length** | -0.035  (-0.201, 0.131) | 0.067 | -2.995 | 0.801  (0.557, 0.917) | 0.829 (*P*<.001) |
|  |  | **Gait speed** | -0.032  (-0.188, 0.124) | 0.063 | -3.078 | 0.844  (0.642, 0.935) | 0.864 (*P*<.001) |
|  | **In-lab walking at fast speed** | **Cadence** | 1.026  (-2.838, 4.889) | 1.322 | 0.810 | 0.975  (0.927, 0.991) | 0.981 (*P*<.001) |
|  |  | **Stride time** | -0.004  (-0.020, 0.012) | 0.006 | -0.405 | 0.994  (0.982, 0.998) | 0.995 (*P*<.001) |
|  |  | **Stride length** | -0.003  (-0.159, 0.154) | 0.054 | -0.394 | 0.897  (0.762, 0.958) | 0.892 (*P*<.001) |
|  |  | **Gait speed** | -0.009  (-0.180, 0.162) | 0.063 | -0.864 | 0.890  (0.749, 0.955) | 0.887 (*P*<.001) |
|  | **In-lab simulated activities** | **Cadence** | 0.385  (-1.230, 1.999) | 0.634 | 0.345 | 0.993  (0.979, 0.997) | 0.994 (*P*<.001) |
|  |  | **Stride time** | -0.006  (-0.017, 0.006) | 0.006 | -0.531 | 0.993  (0.941, 0.998) | 0.997 (*P*<.001) |
|  |  | **Stride length** | -0.018  (-0.146, 0.110) | 0.050 | -1.561 | 0.874  (0.714, 0.948) | 0.879 (*P*<.001) |
|  |  | **Gait speed** | -0.009  (-0.129, 0.112) | 0.048 | -0.833 | 0.912  (0.795, 0.964) | 0.911 (*P*<.001) |
|  |  | **95^th^ percentile of gait speed** | -0.019  (-0.202, 0.164) | 0.069 | -1.597 | 0.859  (0.684, 0.941) | 0.858 (*P*<.001) |
|  | **Outside-lab activities** | **Cadence** | -0.344  (-2.802, 2.115) | 0.516 | -0.299 | 0.989  (0.974, 0.996) | 0.990 (*P*<.001) |
|  |  | **Stride time** | -0.001  (-0.015, 0.012) | 0.003 | -0.119 | 0.998  (0.995, 0.999) | 0.998 (*P*<.001) |
|  |  | **Stride length** | -0.026  (-0.158, 0.106) | 0.051 | -2.051 | 0.879  (0.716, 0.951) | 0.894 (*P*<.001) |
|  |  | **Gait speed** | -0.024  (-0.148, 0.100) | 0.047 | -1.990 | 0.921  (0.810, 0.968) | 0.932 (*P*<.001) |
|  |  | **95^th^ percentile of gait speed** | -0.033  (-0.170, 0.103) | 0.052 | -2.643 | 0.874  (0.687, 0.950) | 0.891 (*P*<.001) |

#### Test-retest reliability of SKDH-gait run on the chest accelerometer

The results of the test-retest reliability analysis over gait endpoints obtained from SKDH-gait run on the APDM chest-worn device across the two visits, performed about 15 days apart, are discussed in this section.

Supplementary Table S4 reports ICC, and Pearson’s R for the test-retest reliability analysis. Similar results for the gait mat are reported in the Supplementary Table S5.

During in-lab walking tasks, ICC shows excellent agreement between all gait endpoints computed during the first visit, and those computed during the second visit, regardless of the self-paced speed. Notably, slightly lower ICC values can be observed for the walking task at slow self-paced speed, both for the APDM chest-worn device, and for the gait mat. This suggests that the lower ICC values can be attributed to the higher variability across visits in the self-paced slow speed, compared to normal and fast self-paced speeds; similarly, keeping a slow pace unchanged during two separate visits might be more challenging, than keeping a normal or a fast pace.

During in-lab simulated activities, ICC and Pearson’s R are even higher for all gait endpoints, as compared to the in-lab walking tasks, ensuring that the APDM chest-worn device can reliably reproduce the same results over time. During outside-lab activities, ICC has a higher variability: it shows an excellent agreement for stride time (ICC=0.766), but a moderate agreement for gait speed (ICC=0.498), and a good agreement for the 95^th^ percentile of gait speed (ICC=0.683). The lower ICC values can be attributed to the different walking behavior across visits, since by task instructions, participants were asked to move freely at any speeds and without pre-specified tasks,

| ***Supplementary Table S4:*** *ICC with LB and UB and Pearson’s R, with their respective p-values between gait endpoints collected from the APDM chest-worn device (SKDH-gait) during the first visit and those collected during the second visit, for all tasks. (LB: lower bound, UB: upper bound)* | | | |
| --- | --- | --- | --- |
| **Task** | **Gait endpoint** | **ICC  (LB, UB)** | **Pearson’s R**  **(p-value)** |
| **In-lab walking at slow speed** | **Cadence** | 0.805  (0.263, 0.937) | 0.888  (*P*<.001) |
|  | **Stride time** | 0.770  (0.156, 0.926) | 0.890  (*P*<.001) |
|  | **Stride length** | 0.861  (0.663, 0.944) | 0.882  (*P*<.001) |
|  | **Gait speed** | 0.761 (0.252, 0.916) | 0.844  (*P*<.001) |
| **In-lab walking at normal speed** | **Cadence** | 0.935  (0.843, 0.974) | 0.942  (*P*<.001) |
|  | **Stride time** | 0.887  (0.734, 0.954) | 0.899  (*P*<.001) |
|  | **Stride length** | 0.828  (0.436, 0.940) | 0.881  (*P*<.001) |
|  | **Gait speed** | 0.802  (0.465, 0.925) | 0.849  (*P*<.001) |
| **In-lab walking at fast speed** | **Cadence** | 0.872  (0.709, 0.947) | 0.879  (*P*<.001) |
|  | **Stride time** | 0.926  (0.825, 0.970) | 0.933  (*P*<.001) |
|  | **Stride length** | 0.970  (0.928, 0.988) | 0.969  (*P*<.001) |
|  | **Gait speed** | 0.934  (0.842, 0.973) | 0.937  (*P*<.001) |
| **In-lab simulated activities** | **Cadence** | 0.912  (0.795, 0.964) | 0.914  (*P*<.001) |
|  | **Stride time** | 0.930  (0.836, 0.972) | 0.928  (*P*<.001) |
|  | **Stride length** | 0.942  (0.860, 0.977) | 0.951  (*P*<.001) |
|  | **Gait speed** | 0.925  (0.824, 0.970) | 0.934  (*P*<.001) |
|  | **95^th^ percentile of gait speed** | 0.914  (0.798, 0.965) | 0.911  (*P*<.001) |
| **Outside-lab activities** | **Cadence** | 0.745  (0.468, 0.890) | 0.751  (*P*<.001) |
|  | **Stride time** | 0.766  (0.505, 0.900) | 0.829  (*P*<.001) |
|  | **Stride length** | 0.565  (0.183, 0.801) | 0.555  (*P*=.011) |
|  | **Gait speed** | 0.498  (0.090, 0.764) | 0.489  (*P*=.028) |
|  | **95^th^ percentile of gait speed** | 0.683  (0.363, 0.860) | 0.671  (*P*=.050) |

| ***Supplementary Table S5:*** *ICC with LB and UB and Pearson’s R, with their respective p-values between gait endpoints collected from the GAITRite gait mat during the first visit and those collected during the second visit, for all in-lab walking tasks. (LB: lower bound, UB: upper bound)* | | | |
| --- | --- | --- | --- |
| **Task** | **Gait endpoint** | **ICC  (LB, UB)** | **Pearson’s R**  **(p-value)** |
| **In-lab walking at slow speed** | **Cadence** | 0.776  (0.044, 0.935) | 0.894  (*P*<.001) |
|  | **Stride time** | 0.809  (0.142, 0.944) | 0.913  (*P*<.001) |
|  | **Stride length** | 0.772  (0.140, 0.928) | 0.871  (*P*<.001) |
|  | **Gait speed** | 0.743 (0.100, 0.917) | 0.856  (*P*<.001) |
| **In-lab walking at normal speed** | **Cadence** | 0.902  (0.747, 0.962) | 0.917  (*P*<.001) |
|  | **Stride time** | 0.885  (0.726, 0.954) | 0.898  (*P*<.001) |
|  | **Stride length** | 0.896  (0.559, 0.966) | 0.935  (*P*<.001) |
|  | **Gait speed** | 0.851 (0.601, 0.943) | 0.882  (*P*<.001) |
| **In-lab walking at fast speed** | **Cadence** | 0.682  (0.361, 0.860) | 0.879  (*P*<.001) |
|  | **Stride time** | 0.931  (0.837, 0.972) | 0.684  (*P*<.001) |
|  | **Stride length** | 0.913  (0.784, 0.965) | 0.938  (*P*<.001) |
|  | **Gait speed** | 0.851  (0.601, 0.943) | 0.891  (*P*<.001) |
